# Radiographic Evaluation of Regeneration Strategies for the Treatment of Advanced Mandibular Furcation Defects: A Retrospective Study

**DOI:** 10.3390/membranes12020219

**Published:** 2022-02-14

**Authors:** Hsiang-Ling Huang, Yun-Han Ma, Che-Chang Tu, Po-Chun Chang

**Affiliations:** 1 Graduate Institute of Clinical Dentistry, School of Dentistry, National Taiwan University, Taipei 10048, Taiwan; r07422019@ntu.edu.tw (H.-L.H.); d05422002@ntu.edu.tw (C.-C.T.); 2 Department of Dentistry, National Taiwan University Hospital, Taipei 10048, Taiwan; b03402008@ntu.edu.tw; 3 School of Dentistry, College of Dental Medicine, Kaohsiung Medical University, Kaohsiung 80708, Taiwan

**Keywords:** periodontal regeneration, furcation defects, barrier membrane, enamel matrix proteins, bone replacement graft

## Abstract

Teeth with furcation involvement (FI) present a higher risk of loss and are difficult to maintain. This study evaluated the efficacy of furcation defect regeneration (FDR) as a regeneration strategy. Pre-operative and 6-month postoperative radiographs were collected from patients receiving regeneration therapy for mandibular teeth with degree II and early degree III FI. The linear furcation involvement (LFI), ratio of LFI (RLI), LFI and RLI adjusted bythe alveolar bone crest (ABC), and radiographic intensity were assessed. The effects of demographic characteristics, regeneration treatment strategies, the relationship between furcation and ABC, and adjacent intrabony defect regeneration (AIDR) were evaluated using a generalized linear model and logistic regression. The results demonstrated that 1.5 mm adjusted LFI and 40% adjusted RLI were achieved in both pure furcation defects and combined furcation–angular defects by the combination of bone replacement grafts (BRG) and enamel matrix derivatives (EMD) or collagen membrane (CM); deproteinized bovine bone matrix (DBBM) showed a superior outcome among BRG. In combined furcation–angular defects, EMD appeared more beneficial than CM, and AIDR significantly promoted adjusted LFI and RLI. In conclusion, DBBM with EMD or CM was effective for FDR, and AIDR had a positive effect on FDR in the combined furcation–angular defect.

## 1. Introduction

Periodontitis is an infection-induced destructive disease affecting 40–70% of the worldwide population, and the prognosis is worse when the furcation area is involved in multi-root teeth [1]. The furcation area represents a complex anatomic morphology that impedes appropriate debridement and instrumentation. Teeth with furcation involvement (FI) exhibit a significantly higher risk of tooth loss for up to 10–15 years even under maintenance care and the increase in risk is associated with the severity of FI [2]. The presence of proximal deep FI also negatively influences the periodontal condition of the neighboring tooth [3]. Treatment options for furcation-involved molars vary according to the severity of FI. Parashis et al. reported that 60–70% of residual calculus remained in the furcation defects after scaling and root planing (SRP), and surgical approaches were more effective, specifically in narrow furcation defects [4]. A systematic review demonstrated that SRP was only effective in teeth with degree I FI, and surgical approaches provided acceptable tooth survival rates (40–100%) for more than 5 years [5]. A recent systematic review further indicated that in teeth with degree II and III FI, the long-term survival rate of surgical resective procedures and SRP was similar [6]. Both reviews also indicated that vertical root fractures, root caries, and endodontic failures could frequently occur in the furcation area following surgical resective procedures.

Periodontal regeneration strategies, including guided tissue regeneration (GTR), bone replacement grafts (BRG), and biomolecules-mediated regeneration, are considered the gold standard for treating teeth with degree II FI, in which the horizontal loss of support exceeds 3 mm but does not encompass the total width of the furcation [7]. Jepsen et al. attempted to establish a ranking of treatment for degree II FI by using Bayesian network meta-analysis and reported that these regeneration strategies generally achieved a substantial 1.3 mm vertical attachment gain and 1.6 mm horizontal attachment gain relative to open flap debridement (OFD) [8]. Due to limited numbers of direct comparisons and included studies, they only concluded that regeneration strategies were superior to OFD, and the supplement of BRG was associated with a higher therapeutic probability and greater clinical improvement [8]. 

This study aimed to directly compare the efficacy of regeneration strategies for treating advanced mandibular FI (degree II or early degree III) in order to establish a protocol for furcation defect regeneration. While collagen membrane (CM), enamel matrix derivatives (EMD), and BRG were the most commonly used materials for periodontal regeneration, to minimize the variables, this study focused on the regeneration strategies of BRG combined with CM or EMD, and the primary outcome was the radiographic bone gain of the furcation defect after 6 months.

## 2. Materials and Methods

### 2.1. Ethical Approval and Case Collection

This retrospective study was approved by the Research Ethics Committee of the National Taiwan University Hospital (NTUH) under a protocol no. 202104011RINA and conducted in compliance with the Declaration of Helsinki of 1975, as amended in 2013 [9]. The charts of patients receiving regeneration treatment for furcation defects of mandibular first and second molars at the Division of Periodontics, Department of Dentistry, NTUH from February 2017 to December 2020 were collected for the analysis.

### 2.2. Inclusion and Exclusion Criteria

The initial records were extracted based on the following criteria.

The inclusion criteria were:

1. The patients were more than 20 years old, without self-reported or pre-diagnosed systemic diseases affecting regeneration outcomes.
2. Comprehensive non-surgical periodontal therapy, including full mouth SRP, and at least two courses of oral hygiene instruction were performed before the regeneration procedure.
3. Degree II or early degree III FI in mandibular molars after the non-surgical periodontal therapy was indicated on the clinical chart.
4. The records of using BRG, including autogenous bone (autograft), freeze-dried bone allograft (FDBA), and deproteinized bovine bone matrix (DBBM), were available on the chart.
5. Radiographic records of furcation defects in the examined teeth before and at least 6 months after the regeneration procedure were available.

The exclusion criteria were:

1. The sole use of BRG without the placement of a CM or EMD.
2. Any fracture or carious lesion reported on the examined teeth before or after the regeneration procedure.
3. Procedures performed on peri-implant tissues or on teeth that were extracted within the next 6 months.
4. Having a history of smoking.

### 2.3. Linear Radiographic Examinations for Furcation Defect Regeneration (FDR)

**Figure 1:**
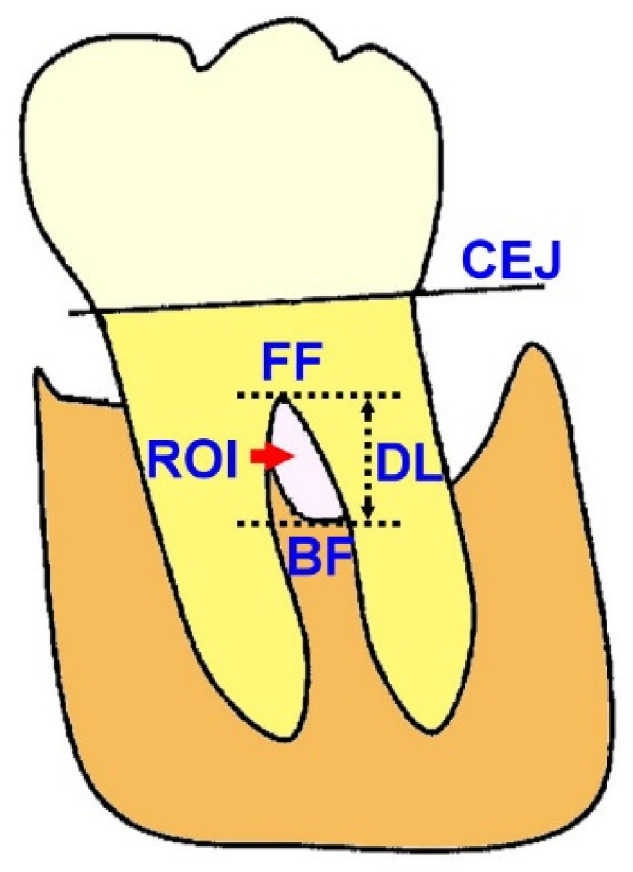
The examined parameters.

All the radiographs were taken in the Oral and Maxillofacial Radiology Unit of NTUH using a dental periapical X-ray machine (Soredex Minray^TM^, Schutterwald, Germany), with the following settings: exposure time 0.63 s; 60 kV for optimized contrast; No. 2-sized film (31 mm × 41 mm) with an X-ray holder (Dentsply International, Inc., Milford, DE, USA). Images were re-oriented to ensure the long axis of the examined teeth was vertically positioned, and the sizes of images at the initial (before the regeneration procedure) and postoperative time points (≥6 months after the regeneration procedure) were normalized based on the width and height of examined teeth on the initial radiographs. The cementoenamel junction (CEJ), fornix of furcation (FF), and the crest at the furcation (BF) of the examined teeth were manually identified, and the defect length (DL) was defined as the distance between FF and BF (Figure 1). 

The following parameters were evaluated.

The linear furcation regeneration improvement (LFI):

(1) $$\mathrm{LFI}=DL_{initial}-DL_{postoperative}$$

The ratio of linear furcation regeneration improvement (RLI):

(2) $$\mathrm{RLI}=\frac{\mathrm{LFI}}{DL_{initial}}\text{×}100\%$$

Both LFI and RLI were further adjusted by the alveolar bone crest (ABC) level across the mesial and distal aspects of the examined teeth, and the adjusted defect length (ADL) was defined as the distance between ABC and BF. The following parameters were evaluated.

The adjusted linear furcation regeneration improvement (ALF):

(3) $$\mathrm{ALF}=ADL_{initial}-ADL_{postoperative}$$

The adjusted ratio of linear furcation regeneration improvement (ARL):

(4) $$\mathrm{ARL}=\frac{\mathrm{ALF}}{ADL_{initial}}\text{×}100\%$$

On the examined teeth with intrabony defects at the mesial or distal aspect, ADL was evaluated based on the most coronal ABC levels across the mesial and distal aspects. The mean length of adjacent intrabony defect resolution (AIDR) was defined as the mean change of distance between the most coronal ABC level and the bottom of the intrabony defect at the mesial and distal aspects of teeth from the initial to postoperative time points.

### 2.4. Radiographic Intensity Measurement of Furcation Defects

The change of radiographic intensity represented the quality of bone formation in the defect region and was investigated by digital subtraction of paired radiographs at different time points [10]. In the present study, the general intensity of images of examined teeth at the initial and postoperative timepoints was normalized based on the grayscale values of surrounding air and dentin and enamel of the examined teeth. On each examined tooth, the area of furcation defect bounded by the tooth root and ABC at the initial time point served as the initial region of interest (ROI) (Figure 1), and the same area at the postoperative time point was chosen as postoperative ROI. The mean grayscale value of ROI at both time points was calculated, and the improvement of radiographic intensity (IRI) was defined as follows:

(5) $$\mathrm{IRI}=\frac{Grayscale_{postoperative}}{Grayscale_{initial}}$$

### 2.5. Statistical Analysis

The data were analyzed using the Statistical Package for the Social Sciences version 25 (IBM Corp., Armonk, NY, USA) and generally presented as mean ± standard deviation (SD). The examined sites were stratified to pure furcation defects and combined furcation–angular defects. The outcome parameters (LFI, RLI, ALF, ARL, and IRI) of treatment strategies were examined using one-way analysis of variance followed by Tukey’s post hoc test (for BRGs) and unpaired t-test (for CM/EMD), with p-values of less than 0.05 considered statistically significant. A generalized linear model (GLM) was used to compare the effects of gender, treatment strategies (CM versus EMD, DBBM versus autografts/FDBA), initial furcation characteristics relative to ABC level, and AIDR, after age adjustment (by the built-in algorithm of the software), and the data were presented as estimates ± SD of the coefficient point estimate. Logistic regression (LR) was used to compare the effects of the demographic characteristics, treatment strategies (CM versus EMD, DBBM versus autografts/FDBA), the extent of initial destruction (degree II FI versus early degree III FI), tooth position (first molar versus the second molar), initial furcation characteristics relative to ABC level, and AIDR in achieving a superior regeneration outcome. The data were presented as the odds ratio (OR) with the range, and the median values of age and outcome parameters were set as the cut-off points.

## 3. Results

### 3.1. Demographic Characteristics and Distribution of Treatment Strategies

A total of 103 furcation-involved teeth, including 37 with pure furcation defects and 66 with combined furcation–angular defects, met the criteria, and all furcation defects were subclass B, which was defined as attachment or bone loss extending to the middle third of the root [11]. The pure furcation defect group comprised 17 female patients and 20 male patients, aged from 33 to 77 years (average 57.38 ± 10.82 years). Of the 37 teeth with furcation defects, 28 were first molars, and 9 were second molars. Thirty teeth were buccal degree II FI, and seven were early degree III FI (mainly involving buccal furcation with slightly perforated lingual furcation). Nine were treated with CM, and twenty-eight were treated with EMD. Autogenous bone was used in 10 teeth, FDBA was used in 4 teeth, and DBBM was used in 23 teeth. The FF was generally 0.69 ± 1.03 mm apical to the ABC and was located coronal to the ABC in seven examined teeth, and the BF was 3.81 ± 1.59 mm apical to ABC. The initial CEJ–BF distance was 6.54 ± 1.79 mm, and the initial furcation DL was 3.12 ± 1.55 mm. No significant difference was observed for the initial ABC–FF, ABC–BF, CEJ–BF, or furcation DL among BRGs or between CM and EMD.

The combined furcation–angular defect group comprised 37 female patients and 29 male patients, aged from 20 to 76 years (average 51.53 ± 10.90 years). Of these 66 teeth, 25 were first molars, and 41 were second molars. Sixty teeth were buccal degree II FI, and six were early degree III FI (mainly involving buccal furcation with slightly perforated lingual furcation). Forty were treated with CM, and the rest were treated with EMD. Autogenous bone was used in 28 teeth, FDBA was used in 15, and DBBM was used in 23. Mesial intrabony defects were visible in 30 teeth (mean intrabony DL was 2.94 ± 1.45 mm), and distal intrabony defects were visible in 38 teeth (mean intrabony DL was 3.60 ± 1.92 mm). Using the most coronal ABC level as a reference, the FF was 1.02 ± 1.15 mm apical to the ABC and located coronal to the ABC in eight examined teeth, and the BF was 3.66 ± 1.48 mm apical to the ABC. The initial CEJ–BF distance was 6.31 ± 1.54 mm, and the initial furcation DL was 2.63 ± 1.31 mm. No significant difference was observed for the initial intrabony DL, ABC–FF, ABC–BF, CEJ–BF, or furcation DL among BRGs or between CM and EMD.

### 3.2. Outcome Analysis: Pure Furcation Defects

**Figure 2:**
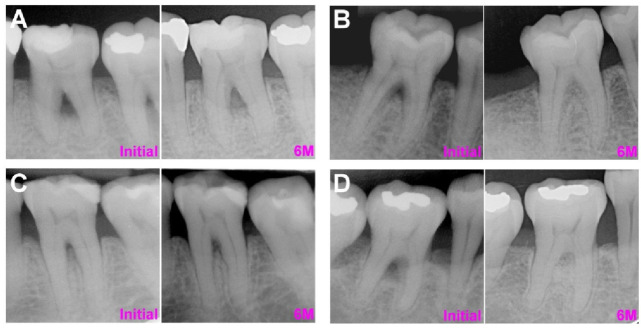
The representative pure furcation defect cases at initial (pre-operative phase) and 6 months following the regenerative procedure. (**A**) Autograft with CM (**B**) DBBM with CM. (**C**) Autograft with EMD. (**D**) DBBM with EMD. *Abbreviations: CM: collagen membrane; DBBM: deproteinized bovine bone matrix; EMD: enamel matrix derivatives*.

In the pure furcation defect group, the overall LFI was 1.45 ± 1.15 mm, the RLI was 50 ± 40%, the ALF was 1.59 ± 1.21 mm, the ARL was 43 ± 31%, and the IRI was 1.05 ± 0.19 (Table 1). The postoperative BF was 1.43 mm coronal to the ABC in one tooth but apical to the ABC in all other teeth. Among BRGs, FDBA and DBBM showed superior regeneration outcomes relative to autograft in all examined parameters. More specifically, the LFI (*p* < 0.01) and RLI (*p* < 0.05) were significantly greater in DBBM relative to autografts, and the IRI was significantly greater in FDBA relative to autografts. Compared with CM-treated sites, the LFI, RFI, and ALF were slightly greater, but the ARL and IRI were slightly reduced in EMD-treated sites. The representative cases of autograft with CM, DBBM with CM, autograft with EMD, and DBBM with EMD are listed in Figure 2.

After age adjustment, the results from GLM demonstrated that DBBM showed significantly greater LFI, RLI, ALF, and ARL relative to autograft or FDBA (Table 2). Significantly superior RLI and ARL were noted in female patients relative to male patients and with CM application relative to EMD. The cut-off values for defining superior regeneration outcomes were >1.53 mm for LFI, >43% for RLI, >1.50 mm for ALF, >40% for ARL, and >1.015 for IRI (Table 3). The LR results demonstrated that the ORs in achieving better regeneration (i.e., greater LFI, RLI, ALF, and ARL) were significantly higher by DBBM relative to autograft or FDBA but lower in younger (<58 years old) and male patients, sites with EMD, and sites with an initial FF–ABC < 0.5 mm. The OR in achieving a better ARL was significantly lower in male patients (Table 3).

### 3.3. Outcome Analysis: Combined Furcation-Angular Defects

**Figure 3:**
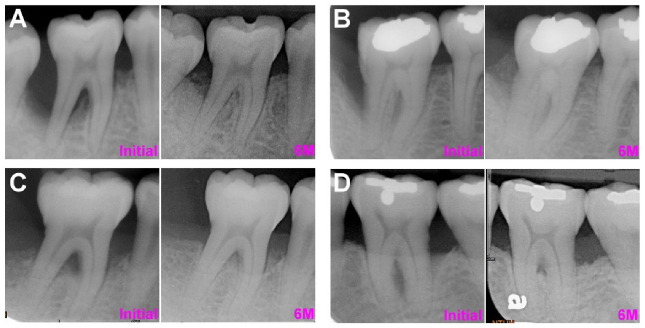
The representative combined furcation-angular defect cases at initial (pre-operative phase) and 6 months following the regenerative procedure. (**A**) FDBA with CM (**B**) DBBM with CM. (**C**) FDBA with EMD. (**D**) DBBM with EMD. *Abbreviations: FDBA: freeze-dried bone matrix; CM: collagen membrane; DBBM: deproteinized bovine bone matrix; EMD: enamel matrix derivatives*.

In the combined furcation–angular defect group, the overall LFI was 1.35 ± 1.25 mm, the RLI was 50 ± 34%, the ALF was 1.41 ± 1.50 mm, the ARL was 34 ± 43%, and the IRI was 1.00 ± 0.16 (Table 4). Using the most coronal ABC as a reference, the postoperative BF was 0.27–2.03 mm coronal to the ABC in five teeth but apical to the ABC in all other teeth. Therefore, using the most apical ABC as a reference, the postoperative BF was 0.20–2.63 mm coronal to the ABC in 10 teeth. Partial adjacent intrabony defect resolution was noted in 41 teeth, and the mean AIDR was 1.57 ± 1.12 mm in these teeth. All examined parameters were insignificantly greater in FDBA and DBBM relative to autografts and insignificantly greater in EMD-treated sites relative to CM-treated sites. The representative cases of autograft with CM, DBBM with CM, autograft with EMD, and DBBM with EMD are listed in Figure 3.

After age adjustment, the results from GLM demonstrated that in sites with detectable AIDR (AIDR > 0 mm), the ALF, ARL, and IRI were significantly improved relative to sites without detectable AIDR (AIDR ≤ 0 mm; Table 5). All examined parameters were improved in DBBM relative to relative to autograft or FDBA except for RLI. In sites with EMD, all examined parameters were insignificantly improved relative to those with CM. The cut-off values for defining superior regeneration outcomes were >1.03 mm for LFI, >40% for RLI, >1.30 mm for ALF, >30% for ARL, and >1.011 for IRI (Table 6). Sites with detectable AIDR also revealed significantly higher ORs in achieving better ALF and ARL outcomes relative to sites without detectable AIDR. Additionally, sites with DBBM and EMD showed higher ORs for all examined parameters in achieving better regeneration relative to sites with autograft or FDBA and CM, respectively.

## 4. Discussion

This study investigated the efficacy of periodontal regeneration strategies in degree II FI based on radiographs. Linear regeneration in the furcation defects was around 1.4 mm or 50%, with an approximate 1% gain of radiographic intensity, regardless of pure furcation defects or combined furcation–angular defects (Table 1 and Table 4). As Horwitz et al. reported, FF coronal to the ABC negatively influenced the regeneration outcome because complete mucosal coverage to allow the colonization of cells within the defect was difficult to achieve [12]. In the present study, the outcome was adjusted by ABC level and showed that an FDR of 1.5 mm or 40% was achieved.

The improvement of FDR using barrier membranes was intensively analyzed. Mechanical instrumentations, including SRP and OFD, have achieved 0.8–1.0 mm vertical clinical attachment gain in the furcation defect [13,14]. A meta-analysis of re-entry data from Kinaia et al. reported that among OFD, non-resorbable membrane, and resorbable membrane in treating mandibular molars with degree II FI, both non-resorbable and resorbable membranes significantly reduced vertical probing depth and vertical bone fill relative to OFD, and resorbable membrane showed superior vertical regeneration outcomes relative to the non-resorbable membrane [15]. Avila-Ortiz et al. summarized the data from 8 clinical trials involving resorbable membrane and OFD and reported that resorbable membrane showed superior clinical outcomes, with >1 mm attachment gain and 70% furcation fill [16]. BRG provides a structural framework and coordinate signals in support of bone formation [17], and the combination of BRG and barrier membrane has been reported as a beneficial approach for FDR [18,19]. Jaiswal et al. reported that an additional 0.6 mm vertical relative attachment level gain was achievable with the combination of BRG and resorbable membrane relative to OFD [18]. Houser et al. showed that DBBM with resorbable membrane contributed to 2.0 mm vertical furcation bone fill, 2.0 mm vertical probing depth reduction, and 82.7% defect resolution in mandibular molars with degree II FI [19]. Data presented in previous studies mostly originated from the clinical soft tissue measurements and might not necessarily correlate to the hard-tissue response, which may be more objectively and accurately assessed by radiographs [20]. The present study analyzed radiographs and concentrated on the hard-tissue parameters in the furcation defects. The results were parallel to these studies and supported that the combination of BRG and resorbable membrane (CM in the present study) improved the FDR. The change of radiographic intensity (i.e., IRI) was also evaluated. The result indicated that IRI mildly increased after regeneration treatment (Table 1 and Table 4), and the data were consistent with previous studies by Avila-Ortiz et al. [16]. Nevertheless, IRI was slightly lower in sites treated with autografts, possibly related to rapid adsorption of the autograft and the transition of BRG to the newly formed bone being incomplete at the time of evaluation.

EMD was introduced as an alternative modality for periodontal regeneration. A multicenter study by Meyle et al. showed that EMD led to similar outcomes of hard-tissue regeneration as the resorbable membrane in teeth with degree II FI [21]. Casarin et al. reported that EMD led to a 1.04 mm vertical bone level gain in degree II FI and converted 73% sites to degree I FI or complete furcation closure [22]. Due to the viscous nature of EMD, although a solid conclusion has not been reached yet, the combination of EMD and BRG has been frequently suggested to secure sufficient space for FDR [23,24]. The data from the present study support that the EMD–BRG combination facilitated FDR (Table 1 and Table 4). Compared with CM, EMD revealed superior regeneration outcomes from the linear measurement. This might relate to the improved mucosal healing following EMD application. As Jepsen et al. indicated, EMD resulted in fewer adverse events and postoperative complications relative to the resorbable membrane and facilitated tissue attachment and the release of growth factors during early healing stages [25]. In a comparative study regarding the intrabony defect, Iorio-Siciliano et al. demonstrated that as space maintenance was provided by BRGs, EMD showed an equivalent outcome of regeneration as resorbable membrane, even in the deep non-contained defects [26]. Because furcation defects are frequently non-contained, and flap management is relatively technically sensitive relative to intrabony defects, the EMD–BRG combination may be an effective approach for FDR.

Among BRG, DBBM and FDBA demonstrated more favorable FDR outcomes relative to autografts (Table 1 and Table 4). Because DBBM was still observed in ≥30% of regenerated or augmented sites after 6 months in previous studies [27,28], the improved regeneration in the present study may partially attribute to the occupation of residual DBBM. Because limited sites were treated by FDBA, the statistical significance of FDBA relative to other BRGs was not achievable. Based on equivalent regeneration outcomes of FDBA and DBBM in the alveolar ridge and sinus augmentation [28,29], a similar regeneration capability of FDBA and DBBM in furcation defects could be expected.

In the combined furcation–angular defect group, the regeneration outcome was similar but slightly inferior to the pure furcation defect group. Therefore, using EMD to replace CM appeared more beneficial in this group, as shown in Table 3 and Table 6. The major reason could be the complexity of combined furcation–angular defects that influenced the proper membrane placement and wound closure and thus compromised the healing process. Notably, the FDR was improved in sites with detectable AIDR (Table 5 and Table 6), suggesting that AIDR had a positive effect on FDR, presumably due to the elevation of the bottom of the ABC that secured more space for cell repopulation [12]. This correlation also inferred the superior inherent regeneration potential of the investigated patients.

The major limitation of this retrospective study was that only radiographs were assessed, and the clinical parameters were not included for the analysis such that changes with clinical significance, including the extent of furcation closure and gingival recession, could not be evaluated. Although radiographic analysis appeared to be a reliable non-invasive method of evaluating clinical bone regeneration, a valid analysis protocol has not been developed yet. Both Toback et al. and Tonetti et al. demonstrated an approximately 1 mm underestimation of clinical bone fill [20,30]; Francis et al. reported that the results from subtraction radiography-based densitometric analysis did not correlate with clinical bone fill [31]. Hence, a combination of radiographic findings with clinical/histological evidence may be necessary to provide a more comprehensive view of FDR. On the other hand, radiographies at 6 months post-operatively were assessed because the increase of the radiographic intensity can be visualized. As Rakmanee et al. reported a substantial improvement on radiographies from 6 to 12 months [32], the follow-up period of this study was relatively short such that the improvement on the radiography might be underestimated. However, this is the first study to directly compare the efficacy among BRGs and between CM/EMD specifically in the furcation defects and still provide valuable insight for establishing an appropriate protocol for FDR. Further studies with the combination of clinical assessments, re-entry measurements, and histological evidence, with a longer follow-up period, are still required.

## 5. Conclusions

This radiography-based study supported that combining BRG with EMD or CM promoted FDR. Among BRGs, DBBM appeared to show more favorable regeneration outcomes. In combined furcation–angular defects, using EMD to replace CM was beneficial, and AIDR revealed a positive effect in facilitating FDR.

## Figures and Tables

**Table 1:** The outcome of FDR in the pure furcation defect group based on treatment strategies.

		LFI		RLI		ALF		ARL		IRI	
		Mean ± SD	*p*	Mean ± SD	*p*	Mean ± SD	*p*	Mean ± SD	*p*	Mean ± SD	*p*
Overall		1.45 ± 1.15 mm		50 ± 40%		1.59 ± 1.21 mm		43 ± 31%		1.05 ± 0.19	
BRG	Autograft	0.68 ± 1.00 mm	0.039 *	16 ± 25%	0.003 **	1.00 ± 1.10 mm	0.195	28 ± 28%	0.185	0.98 ± 0.15	0.043 *
	FDBA	1.77 ± 1.08 mm		52 ± 34%		1.78 ± 1.89 mm		57 ± 66%		1.25 ± 0.26	
	DBBM	1.74 ± 1.11 mm ^††^		64 ± 38% ^†^		1.82 ± 1.09 mm		47 ± 23%		1.04 ± 0.17	
	CM	1.27 ± 1.03 mm	0.589	38 ± 36%	0.287	1.58 ± 1.31 mm	0.967	52 ± 44%	0.321	1.10 ± 0.26	0.394
	EMD	1.52 ± 1.20 mm		54 ± 41%		1.60 ± 1.20 mm		40 ± 26%		1.02 ± 0.25	

Abbreviations: LFI: the linear furcation regeneration improvement; RLI: the ratio of LFI; ALF: the adjusted LFI; ARL: the adjusted ratio of LFI; IRI: the improvement of radiographic intensity; BRG: the bone replacement graft; FDBA: freeze-dried bone matrix; DBBM: deproteinized bovine bone matrix; CM: collagen membrane; EMD: enamel matrix derivatives. Significant difference in the strategy: * *p* < 0.05, ** *p* < 0.01; Significant difference to autograft treatment: ^†^ *p* < 0.05, ^††^ *p* < 0.01.

**Table 2:** Generalized linear model for the effects of the demographic characteristics, treatment strategies, and furcation-ABC relationship, on the outcome of FDR in the pure furcation defect group. The data was adjusted by age.

	LFI		RLI		ALF		ARL		IRI	
	Estimate ± SD	*p*	Estimate ± SD	*p*	Estimate ± SD	*p*	Estimate ± SD	*p*	Estimate ± SD	*p*
Male vs. female	−0.61 ± 0.37	0.100	−27.7 ± 11.2%	0.013 *	−0.50 ± 0.40	0.209	−23.1 ± 9.48%	0.015 *	−0.01 ± 0.07	0.930
DBBM vs. autograft/FDBA	1.16 ± 0.46	0.011 *	54.1 ± 13.9%	0.000 ***	1.08 ± 0.50	0.029 *	35.6 ± 11.8%	0.003 **	0.02 ± 0.08	0.817
EMD vs. CM	−0.79 ± 0.54	0.143	−33.0 ± 16.4%	0.044 *	−0.89 ± 0.58	0.126	−40.6 ± 13.8%	0.003 **	−0.08 ± 0.09	0.406
FF-ABC ≥ 0.5 mm vs. <0.5 mm	0.16 ± 0.36	0.665	12.3 ± 11.1%	0.267	0.07 ± 0.39	0.867	−7.80 ± 9.36%	0.404	0.06 ± 0.07	0.358

Abbreviations: LFI: the linear furcation regeneration improvement; RLI: the ratio of LFI; ALF: the adjusted LFI; ARL: the adjusted ratio of LFI; IRI: the improvement of radiographic intensity; BRG: the bone replacement graft; FDBA: freeze-dried bone matrix; DBBM: deproteinized bovine bone matrix; CM: collagen membrane; EMD: enamel matrix derivatives; FF-ABC: the distance between FF and ABC. Significant difference: * *p* < 0.05, ** *p* < 0.01, *** *p* < 0.001.

**Table 3:** Logistic regression for the effects of the demographic characteristics, treatment strategies, and furcation–ABC relationship in achieving a better outcome of FDR in the pure furcation defect group.

	LFI > 1.53 mm		RLI > 43%		ALF > 1.50 mm		ARL > 40%		IRI > 1.015	
	OR [Range]	*p*	OR [Range]	*P*	OR [Range]	*p*	OR [Range]	*p*	OR [Range]	*p*
≥ 58 year-old vs. <58 year-old	5.46 [0.97–30.68]	0.054	1.24 [0.23–6.56]	0.802	4.22 [0.82–21.84]	0.086	1.23 [0.26–5.98]	0.788	0.54 [0.13–2.30]	0.541
Male vs. female	0.42 [0.08–2.24]	0.307	0.20 [0.03–1.30]	0.092	0.43 [0.08–2.38]	0.334	0.11 [0.02–0.77]	0.025 *	0.77 [0.17–3.51]	0.738
DBBM vs. autograft/FDBA	24.42 [1.77–336.26]	0.017 *	13.86 [1.50–128.58]	0.021 *	11.50 [1.26–105.15]	0.031 *	14.87 [1.46–151.54]	0.023 *	0.66 [0.10–4.23]	0.660
EMD vs. CM	0.13 [0.01–2.24]	0.162	0.66 [0.06–7.67]	0.737	0.23 [0.02–2.75]	0.243	0.18 [0.02–2.15]	0.174	1.13 [0.14–9.27]	0.908
Degree II FI vs. degree III FI	0.32 [0.03-4.11]	0.384	0.09 [0.01–1.24]	0.072	0.16 [0.01–1.84]	0.141	0.14 [0.01–1.53]	0.108	2.26 [0.29–17.57]	0.436
First molar vs. second molar	6.83 [0.69-67.27]	0.100	2.10 [0.25–17.38]	0.492	2.04 [0.29–14.14]	0.472	2.49 [0.36–17.06]	0.353	3.60 [0.55–23.57]	0.182
FF-ABC ≥ 0.5 mm vs. <0.5 mm	2.51 [0.45-13.99]	0.295	2.58 [0.50–13.44]	0.259	2.00 [0.39–10.21]	0.404	0.63 [0.12–3.29]	0.585	2.09 [0.48–9.04]	0.325

Abbreviations: LFI: the linear furcation regeneration improvement; RLI: the ratio of LFI; ALF: the adjusted LFI; ARL: the adjusted ratio of LFI; IRI: the improvement of radiographic intensity; BRG: the bone replacement graft; FDBA: freeze-dried bone matrix; DBBM: deproteinized bovine bone matrix; CM: collagen membrane; EMD: enamel matrix derivatives; FF-ABC: the distance between FF and ABC. Significant difference: * *p* < 0.05.

**Table 4:** The outcome of FDR in the combined furcation-angular defect group based on treatment strategies.

		LFI		RLI		ALF		ARL		IRI	
		Mean ± SD	*p*	Mean ± SD	*p*	Mean ± SD	*p*	Mean ± SD	*p*	Mean ± SD	*p*
Overall		1.35 ± 1.25 mm		50 ± 34%		1.41 ± 1.50 mm		34 ± 43%		1.00 ± 0.16	
BRG	Autograft	1.22 ± 1.33 mm	0.661	51 ± 37%	0.939	1.23 ± 1.64 mm	0.406	29 ± 49%	0.424	0.97 ± 0.11	0.445
	FDBA	1.32 ± 1.37 mm		47 ± 36%		1.47 ± 1.57 mm		36 ± 38%		1.02 ± 0.22	
	DBBM	1.54 ± 1.09 mm		50 ± 31%		1.61 ± 1.29 mm		39 ± 37%		1.03 ± 0.18	
	CM	1.21 ± 1.36 mm	0.257	46 ± 35%	0.257	1.34 ± 1.67 mm	0.632	32 ± 43%	0.615	0.97 ± 0.14	0.088
	EMD	1.57 ± 1.05 mm		56 ± 34%		1.52 ± 1.21 mm		37 ± 43%		1.05 ± 0.18	

Abbreviations: LFI: the linear furcation regeneration improvement; RLI: the ratio of LFI; ALF: the adjusted LFI; ARL: the adjusted ratio of LFI; IRI: the improvement of radiographic intensity; BRG: the bone replacement graft; FDBA: freeze-dried bone matrix; DBBM: deproteinized bovine bone matrix; CM: collagen membrane; EMD: enamel matrix derivatives.

**Table 5:** Generalized linear model for the effects of the demographic characteristics, treatment strategies, furcation–ABC relationship, and AIDR on the outcome of FDR in the combined furcation–angular defect group. The data was adjusted by age.

	LFI		RLI		ALF		ARL		IRI	
	Estimate ± SD	*p*	Estimate ± SD	*p*	Estimate ± SD	*p*	Estimate ± SD	*p*	Estimate ± SD	*p*
Male vs. female	0.12 ± 0.30	0.690	8.20 ± 8.58%	0.340	−0.02 ± 0.34	0.966	1.20 ± 10.08%	0.908	−0.03 ± 0.04	0.406
DBBM vs. autograft/FDBA	0.21 ± 0.32	0.508	−2.00 ± 9.13%	0.823	0.23 ± 0.37	0.537	5.80 ± 10.72%	0.586	0.00 ± 0.04	0.941
EMD vs. CM	0.30 ± 0.31	0.328	9.70 ± 8.96%	0.278	0.21 ± 0.36	0.563	6.10 ± 10.52%	0.565	0.07 ± 0.04	0.110
FF-ABC ≥ 0.5 mm vs. <0.5 mm	−0.43 ± 0.33	0.195	−4.10 ± 9.54%	0.665	−0.06 ± 0.38	0.882	1.30 ± 11.21%	0.905	0.06 ± 0.05	0.232
BF-ABC ≥ 0.5 mm vs. <0.5 mm	−0.49 ± 0.42	0.248	−0.80 ± 12.23%	0.950	−0.19 ± 0.49	0.701	−3.20 ± 14.37%	0.822	0.02 ± 0.05	0.685
AIDR > 0 mm vs. AIDR ≤ 0 mm	0.42 ± 0.31	0.181	9.10 ± 9.01%	0.311	1.23 ± 0.36	0.001 **	30.3 ± 5.10%	0.004 **	0.10 ± 0.04	0.027 *

Abbreviations: LFI: the linear furcation regeneration improvement; RLI: the ratio of LFI; ALF: the adjusted LFI; ARL: the adjusted ratio of LFI; IRI: the improvement of radiographic intensity; BRG: the bone replacement graft; FDBA: freeze-dried bone matrix; DBBM: deproteinized bovine bone matrix; CM: collagen membrane; EMD: enamel matrix derivatives; FF-ABC: the distance between FF and ABC; AIDR: the adjacent intrabony defect regeneration. Significant difference: * *p* < 0.05, ** *p* < 0.01.

**Table 6:** Logistic regression for the effects of the demographic characteristics, treatment strategies, furcation–ABC relationship, and AIDR in achieving a better outcome of FDR in the combined furcation–angular defect group.

	LFI > 1.03 mm		RLI > 40%		ALF > 1.30 mm		ARL > 30%		IRI > 1.011	
	OR [Range]	*p*	OR [Range]	*p*	OR [Range]	*p*	OR [Range]	*p*	OR [Range]	*p*
≥ 52 year-old vs. < 52 year-old	1.66 [0.48–5.73]	0.424	0.46 [0.15–1.39]	0.168	1.03 [0.32–3.29]	0.965	0.54 [0.18–1.68]	0.290	1.11 [0.30–4.13]	0.877
Male vs. female	2.73 [0.79–9.37]	0.111	1.60 [0.55–4.64]	0.387	0.65 [0.21–2.03]	0.456	0.95 [0.32–2.84]	0.930	1.47 [0.41–5.29]	0.558
DBBM vs. autograft/FDBA	1.79 [0.52–6.15]	0.357	1.40 [0.44–4.43]	0.565	1.62 [0.49–5.37]	0.430	1.18 [0.37–3.80]	0.778	1.33 [0.39–4.53]	0.653
EMD vs. CM	2.73 [0.78–9.61]	0.118	1.68 [0.56–5.04]	0.357	1.58 [0.49–5.11]	0.447	1.83 [0.59–5.69]	0.297	2.20 [0.63–7.65]	0.217
Degree II FI vs. degree III FI	NA	NA	1.95 [0.24–15.99]	0.535	NA	NA	NA	NA	NA	NA
First molar vs. second molar	0.58 [0.13-2.57]	0.475	0.41 [0.12–1.41]	0.156	0.36 [0.09–1.38]	0.136	0.52 [0.15–1.83]	0.308	1.41 [0.43–4.62]	0.573
FF-ABC ≥ 0.5 mm vs. <0.5 mm	0.87 [0.23-3.33]	0.835	0.60 [0.17–2.10]	0.421	1.08 [0.29–4.02]	0.906	0.86 [0.24–3.10]	0.816	2.99 [0.64–14.01]	0.166
BF-ABC ≥ 0.5 mm vs. <0.5 mm	0.13 [0.02-1.06]	0.057	1.36 [0.30–6.21]	0.693	0.86 [0.17–4.23]	0.849	1.58 [0.34–7.38]	0.558	0.44 [0.10–2.00]	0.288
AIDR > 0 mm vs. AIDR ≤0 mm	2.98 [0.90-9.82]	0.073	1.70 [0.55–5.26]	0.357	5.13 [1.56–16.84]	0.007 **	3.79 [1.18–12.15]	0.025 *	1.64 [0.48–5.56]	0.432

Abbreviations: LFI: the linear furcation regeneration improvement; RLI: the ratio of LFI; ALF: the adjusted LFI; ARL: the adjusted ratio of LFI; IRI: the improvement of radiographic intensity; BRG: the bone replacement graft; FDBA: freeze-dried bone matrix; DBBM: deproteinized bovine bone matrix; CM: collagen membrane; EMD: enamel matrix derivatives; FF-ABC: the distance between FF and ABC; AIDR: the adjacent intrabony defect regeneration; NA: not applicable due to very limited pairs for the comparison. Significant difference: * *p* < 0.05, ** *p* < 0.01.

## Data Availability

The data that support the findings of this study are available from the corresponding author upon reasonable request.
